# NEIGHBORHOOD CHARACTERISTICS AND SLEEP PROBLEMS: MEDIATING ROLES OF HEALTH BEHAVIORS AND MENTAL HEALTH

**DOI:** 10.1093/geroni/igad104.2374

**Published:** 2023-12-21

**Authors:** Ethan Siu Leung Cheung

**Affiliations:** University of Utah, Salt Lake City, Utah, United States

## Abstract

Existing studies have well-documented the relationship between the neighborhood environment and health among older adults. Yet very few have focused on how the neighborhood environment predicts late-life sleep quality. Using longitudinal data (Rounds 7-9) from the National Health and Aging Trends Study, this study examined the cross-sectional and longitudinal associations between neighborhood characteristics (physical disorder and social cohesion) and sleep problems among Americans aged 65 or older, as well as whether health behaviors and mental health mediated these associations. The analytical sample included 4,029 adults aged 65 or older. Logistic regressions with a lagged dependent variable were adopted to test longitudinal associations. Longitudinal mediation analyses were also used to examine the mediation effects of health behaviors and mental health. The results suggested that neighborhood physical disorder was a significant predictor of sleep problems cross-sectionally, whereas social cohesion was both cross-sectionally and longitudinally associated with sleep problems. Moreover, mediation analysis revealed that health behaviors and mental health significantly mediated the longitudinal relationship between social cohesion and sleep problems, though the strength of the indirect effect diminished over time. These findings suggest that neighborhood physical disorder and social cohesion are significant predictors of late-life sleep problems among older Americans. The study also highlighted older adults’ resilience and adaptability to adverse neighborhood environments and the long-term health benefits of social cohesion in late-life sleep quality. Therefore, community health interventions that facilitate older adults’ physical and social activity, along with mental health programs, may help community-dwelling older adults improve their sleep quality.

